# 

**Published:** 1853-08

**Authors:** 


### CONTENTS OF AUGUST NO.

Sanborn on Medical Education,.......... Page 1
McGugin on Erysipelas,.......... 8
Hall on Spinal Irritation,.......... 13
Washington on Dry Cupping,.......... 15
Editorial Salutatory,.......... 17
Dr. Hughes' Letter,.......... 20
Medical Department of Iowa University,.......... 27
Changes in Medical Faculty,.......... 29
State Medical Society.......... 30
Notice to Subscribers,.......... 32

